# Plasmatic Oxidative and Metabonomic Profile of Patients with Different Degrees of Biliary Acute Pancreatitis Severity

**DOI:** 10.3390/antiox10060988

**Published:** 2021-06-21

**Authors:** Pedro Silva-Vaz, Ivana Jarak, Luís Rato, Pedro F. Oliveira, Sara Morgado-Nunes, Aida Paulino, Miguel Castelo-Branco, Maria Filomena Botelho, José Guilherme Tralhão, Marco G. Alves, Ana Margarida Abrantes

**Affiliations:** 1 Health Sciences Research Centre, University of Beira Interior (CICS-UBI), 6200-506 Covilhã, Portugal; mcbranco@fcsaude.ubi.pt; 2 General Surgery Department, Hospital Amato Lusitano, Unidade Local de Saúde de Castelo Branco, 6000-085 Castelo Branco, Portugal; apaulino@ulscb.min-saude.pt; 3 Faculty of Health Sciences, University of Beira Interior, 6200-506 Covilhã, Portugal; 4 Clinical Academic Centre of Beiras (CACB), 6200-506 Covilhã, Portugal; sara@ipcb.pt; 5 Department of Pharmaceutical Technology, Faculty of Pharmacy, University of Coimbra, 3000-548 Coimbra, Portugal; uc40924@uc.pt; 6 Health School of the Polytechnic of Guarda, 6300-559 Guarda, Portugal; lrato@ipg.pt; 7 QOPNA & LAQV, Department of Chemistry, University of Aveiro, 3810-193 Aveiro, Portugal; p.foliveira@ua.pt; 8 Polytechnic Institute of Castelo Branco, Escola Superior de Gestão, 6000-084 Castelo Branco, Portugal; 9 Biophysics Institute, Faculty of Medicina, University of Coimbra, 3000-548 Coimbra, Portugal; mfbotelho@fmed.uc.pt (M.F.B.); jgtralhao@fmed.uc.pt (J.G.T.); mabrantes@fmed.uc.pt (A.M.A.); 10 Coimbra Institute for Clinical and Biomedical Research (iCBR) Area of Environment Genetics and Oncobiology (CIMAGO), Faculty of Medicina, University of Coimbra, 3000-548 Coimbra, Portugal; 11 CNC.IBILI Consortium/Center for Innovation Biomedicine and Biotechnology (CIBB), University of Coimbra, 3000-548 Coimbra, Portugal; 12 Clinical Academic Center of Coimbra (CACC), 3000-561 Coimbra, Portugal; 13 Surgery Department, Centro Hospitalar e Universitário de Coimbra (CHUC), Faculty of Medicina, University Hospital, 3000-075 Coimbra, Portugal; 14 Department of Anatomy and Unit for Multidisciplinary Research in Biomedicine (UMIB), Institute of Biomedical Sciences Abel Salazar (ICBAS), University of Porto, 4050-313 Porto, Portugal; maalves@icbas.up.pt

**Keywords:** acute biliary pancreatitis, inflammation, prognostic, severity, hepcidin, systemic inflammatory response index, oxidative stress, metabonomics

## Abstract

Acute pancreatitis (AP) is an inflammatory process of the pancreas with variable involvement of the pancreatic and peripancreatic tissues and remote organ systems. The main goal of this study was to evaluate the inflammatory biomarkers, oxidative stress (OS), and plasma metabolome of patients with different degrees of biliary AP severity to improve its prognosis. Twenty-nine patients with biliary AP and 11 healthy controls were enrolled in this study. We analyzed several inflammatory biomarkers, multifactorial scores, reactive oxygen species (ROS), antioxidants defenses, and the plasma metabolome of biliary AP and healthy controls. Hepcidin (1.00), CRP (0.94), and SIRI (0.87) were the most accurate serological biomarkers of AP severity. OS played a pivotal role in the initial phase of AP, with significant changes in ROS and antioxidant defenses relating to AP severity. Phenylalanine (*p* < 0.05), threonine (*p* < 0.05), and lipids (*p* < 0.01) showed significant changes in AP severity. The role of hepcidin and SIRI were confirmed as new prognostic biomarkers of biliary AP. OS appears to have a role in the onset and progression of the AP process. Overall, this study identified several metabolites that may predict the onset and progression of biliary AP severity, constituting the first metabonomic study in the field of biliary AP.

## 1. Introduction

Acute pancreatitis (AP) is an inflammatory disorder of the exocrine pancreas with high morbidity and mortality if associated with local and systemic complications [1,2]. Most patients present with mild self-limited AP with a good prognosis [3]. However, 15–20% progress to a severe AP with pancreatic necrosis and high morbidity and mortality associated with systemic inflammatory response syndrome (SIRS) and multiple organ failure [4,5]. There has been an increasing incidence in the last decades, although with an unchanged mortality rate [6]. The overall mortality of AP is less than 5%, though severe AP is associated with a mortality up to 30–50% [1,7]. The most common causes of AP are gallstones and alcohol [6]. In the Mediterranean and other southern European countries, including Portugal, gallstones are the dominant etiology [8].

Several theories associated with the early pathogenesis of AP have been described, such as the intra-acinar and ductal activation of proteolytic enzymes, leukocyte chemotaxis, the release of pro- and anti-inflammatory cytokines, oxidative stress (OS), mitochondrial dysfunction, gallstone migration, microcirculation injury, as well as bacterial translocation to the pancreas and systemic circulation [9,10]. Nevertheless, the molecular mechanisms by which those effects are mediated remain unknown.

AP is associated with a high variability of severity, and although an early identification of patients who will develop severe AP is crucial, this identification remains a great challenge. Determination of AP prognosis is crucial to optimize the initial therapeutic approach and decrease mortality. Several serological biomarkers and scores have been described, namely C-reactive protein (CRP) [11], hepcidin [12,13], procalcitonin (PCT) [14], the systemic inflammatory response index (SIRI) [13], the bedside index for severity in acute pancreatitis (BISAP) [15], SIRS [16], and the modified Marshall score (MMS) [17].

The role of reactive oxygen species (ROS) in the pathophysiology of AP has been demonstrated [18,19] and occurs in the early stages of this inflammatory process [20]. However, it is not clear whether ROS act as mediators or initiate the complex cascade that leads to AP and, thus, the exact role of OS in the development of AP remains unclear [21]. OS is presently considered a critical mediator of the early local events related to AP and the associated SIRS [22]. Mitochondria are susceptible to OS, and mitochondrial dysfunction is often used as a specific biomarker of oxidant exposure [23]. In experimental models of AP, acinar cells were shown to die through necrosis and apoptosis, pathways linked to mitochondrial dysfunction [24]. Others suggest that mitochondrial dysfunction is associated with systemic inflammatory processes [25], but the mechanisms remain largely unknown. Thus, OS-associated events and biomarkers in AP remain to be defined.

The determination of metabolite changes that describe a biological phenotype based on ^1^H nuclear magnetic resonance (NMR) spectroscopy has been widely applied to define prognostic biofluid profiles for physiological or pathological states [26]. In inflammatory disorders, such as AP, only a few studies of metabolome have been reported. Villaseñor et al. [27] studied the metabolic phenotype in urine and plasma samples of 15 patients with AP. The etiologies of the AP group were gallstones (6/15) and alcohol (9/15). They found changes in alanine, valine, and hippurate in patients with AP and concluded that these metabolites have the potential to serve as diagnostic tools. Lusczek et al. [28] evaluated the potential of urinary ^1^H-NMR metabonomics in the diagnosis of AP. The authors analyzed five patients with AP and concluded that it is possible to obtain a distinct metabolome in AP patients’ urine samples compared to healthy controls. That study identified citrate as the primary metabolite associated with the inflammatory AP state and alcohol consumption. Xu et al. [29] studied the serum metabolomics of mild AP in 38 patients. Although the authors did not specify the etiology of AP patients, by using the chromatography-high-resolution mass spectrometry (UPLC-HRMS) technique, they identified decanyl choline, dodecanol, and 2-tetradecanone as diagnosis metabolites, and sphinganine, L-thyronine, glycocholic acid and 2-tetradecanone as therapeutic response metabolites. Xiao et al. [30], using gas-chromatography/mass spectrometry (GS-MS), identified several metabolites, such as 3-hydroxubutyric, D-glucose, and hexadecenoic acid, with potential clinical relevance for diagnosis in 40 patients with AP. It was suggested 3-hydroxybutyric acid and citric acid have a prognosis value. Huang et al. [31] studied three AP etiologies (29 patients with hyperlipidemia, 20 with alcoholic AP, and 27 with biliary AP). They concluded that L-tyrosine, octadecanoic acid, cholesterol, glycerol 1-hexadecanoate, and L-lactic acid are metabolites with potential diagnosis relevance and (R)-3-hydroxybutyric acid and mannitol could distinguished AP from the healthy group. However, this study did not evaluate the characteristics between each group of AP patients, nor was the prognosis of AP severity taken into account. These five papers described several metabolites, mainly as diagnostic tools, but only one specifically studied the subgroup of patients with biliary AP, comparing it with other AP etiologies.

This study aimed to evaluate the pathophysiology of AP by analyzing the inflammatory biomarkers and multifactorial scores, OS, and plasma metabolome of biliary AP patients and healthy controls. It is intended to contribute to a better understanding of biliary AP’s pathophysiology and thus improve prognosis.

## 2. Materials and Methods

### 2.1. Participants

Study participants were recruited among patients with biliary AP admitted to the Department of General Surgery of the Hospital Amato Lusitano of Unidade Local de Saúde de Castelo Branco, University Teaching Hospital, Castelo Branco, Portugal. Twenty-nine patients and 11 healthy controls were considered for the study. The study protocol was approved by the local ethics committee (reference number 10294/15) and was conducted in compliance with the Declaration of Helsinki. Signed informed consent was obtained from all participants.

### 2.2. Study Design

Subjects with AP that accepted the request to participate and met the eligibility criteria of admission were recruited between November 2015 and January 2016. AP was defined according to the revised Atlanta classification (RAC). At least two of the following three features must have been present: (1) abdominal pain consistent with AP; (2) serum lipase activity (or amylase activity) at least three times greater than the upper limit of a normal value; (3) and characteristics findings of AP on contrast-enhanced computed tomography (CECT) and less commonly on magnetic resonance imaging (MRI) or transabdominal ultrasonography (US) [17].

All adult patients (≥18 years old) with biliary AP were included. Patients with other causes of AP except biliary, recurrent AP, chronic pancreatitis, pancreatitis due to malignancy, pregnant patients, patients with time from onset of disease to presentation in the emergency room greater than 24 h, and patients being hospitalized for more than 24 h at the time of recruitment were excluded. For an etiological confirmation of AP, the patients underwent US with identification of gallstones or microlithiasis. After the US, patients with etiological doubt underwent endoscopic ultrasonography to exclude microlithiasis and characterize the biliary tree.

Local and systemic complications were defined according to the RAC [17]. The severity of AP was defined according to the RAC: mild (no organ failure or no local or systemic complications), moderately severe (organ failure that resolves within 48 h and the present local or systemic complications or both without persistent organ failure), and severe (presence of persistent organ failure or SIRS). Overall, this study included 40 individuals: mild (*n* = 10), moderately severe (*n* = 9), and severe (*n* = 10) AP patients and healthy controls (*n* = 11).

All subjects included in this study were evaluated for comorbidities by applying the Charlson comorbidity index (CCI). This index is the most frequently used tool to measure co-existing diseases and it has been validated for predicting the risk of mortality, disability, hospitalization, and length of hospital stay in several clinical settings [32]. CCI is age-dependent and evaluates several pathological conditions, including cardiovascular, respiratory, liver, and peptic diseases as well as diabetes and AIDS [33].

### 2.3. Analysis of Biomarkers and Scores Systems

Blood samples were collected from each patient that had been fasting for at least 6 h on admission and after 48 h of onset of symptoms, drawn into 5 mL heparin-treated tubes and centrifuged for 10 min at 1500× *g*. The plasma was then collected and stored at −80 °C until analysis. Lipase (U/L), amylase (U/L), CRP (mg/dL9, PCT (ng/dL), white blood cells (WBC, 10^3^/µL), neutrophil (N, 10^3^/µL), lymphocyte (L, 10^3^/µL), monocyte (M, 10^3^/µL), calcium (mg/dL), albumin (g/dL), total proteins (g/dL), and hepcidin (ng/mL) were analyzed following certified analysis at the Department of Clinical Pathology, Hospital Amato Lusitano of the Unidade Local de Saúde de Castelo Branco. Neutrophil/lymphocyte (N/L) ratio and SIRI were calculated.

The BISAP score was described in 2008 and consists of a simple, early assessment of mortality risk in patients with AP. It evaluated five parameters: blood urea nitrogen (BUN), mental status, SIRS, age, and pleural effusion. The cut-off used to assess the mortality risk and severity of AP was ≥3. SIRS is a simple and widely used score in a clinical setting. It evaluates four parameters: temperature, respiratory rate, pulse, and WBCs. This score is defined by the presence of ≥2 of the described parameters. The MMS allows for the assessment of multiorgan failure in AP. Organ failures includes one or more of the following parameters: cardiovascular, respiratory, and kidney failure. Multiorgan failure is present to a cut-off of ≥2. In the present study, all three scores were assessed on admission and 48 h after onset of symptoms.

### 2.4. Oxidative Stress Evaluation

Carbonyl groups, protein nitration, and lipid peroxidation are usually used as biomarkers for OS-related alterations in proteins and lipids. We evaluated, on admission: 2,4-dinitrophenyl (2,4-DNP), 3-nitrotyrosine (3-NO), and 4-hydroxynonenal (4-HNE), respectively. The content of these adducts in plasma samples was evaluated using specific antibodies via slot-blot as previously described [34,35]. The activity of several antioxidant defense enzymes was also evaluated, namely glutathione peroxidase (GPx), glutathione reductase (GR), superoxide dismutase (SOD), and catalase (CAT). GPx and GR concentrations in plasma samples were determined using previously described methods [36]. The assay for SOD activity was based on the reaction in which SOD reduces the superoxide anion to hydrogen peroxide and oxygen, using a method previously described [37]. The CAT activity assay was based on the measurement of hydrogen peroxide produced by the action of CAT.

### 2.5. Proton NMR (^1^H-NMR) Spectroscopy

^1^H-NMR spectra of plasma samples were acquired and quantified as previously described [38]. Sodium fumarate (final concentration of 1 mM) was used as an internal reference (6.50 ppm) to quantify the following metabolites present in plasma samples media (multiplet, ppm). Relative areas of ^1^h-NMR resonances and metabolites concentrations were quantified as previously described [38]. In brief, plasma samples composed of patients with biliary AP, collected on admission, and healthy subjects for the control group were analyzed. These samples were thawed, homogenized using a vortex, and centrifuged (9200 rpm, 5 min). NMR spectra were acquired on a Varian Inova 600 MHz (14.1 T) spectrometer equipped with a 3 mm QXI probe with a z-water presaturation. Water saturation frequencies were optimized for each sample. Chemical shifts were internally referenced to a glucose doublet at 5.23 ppm. Metabolite assignment was based on a comparison with previously published data and reference spectra available in public databases such as HMBD [39]. Additionally, 2D homonuclear TOCSY spectra were recorded to help with spectral assignment [40].

Data matrices for multivariate analysis were created in AMIX-Viewer (version 3.9.15, BrukerBiospin, Rheinstetten) using all intensity values in the 0.75–8.5 ppm region with the exclusion of water and fumarate regions as well as the regions without signal. Processed spectra were aligned to minimize shift variations [41] and normalized by integral area to account for matrix dilution effects and experimental conditions [42]. Multivariate analysis was performed in Simca 14 (Umetrics, Umea, Sweden) on unit-variance scaled data matrices. Principal component analysis (PCA) evaluated the initial data structure, followed by partial least squares discriminant analysis (PLS-DA) to identify metabolite contribution to class separation. PLS-DA loadings were calculated by multiplying the variable weights (w) with the respective standard deviations and color-coded according to the size of variable importance to projection values (VIP). Metabolites with VIP > 1 were considered relevant to group separation. Default sevenfold cross-validation and permutation tests [43] were used to validate the observed variation (R^2^) and predictive potential (Q^2^) of PLS-DA models. Additionally, well-resolved peaks of relevant metabolites were integrated (AMIX-Viewer, version 3.9.15, BrukerBiospin, Rheinstetten) and normalized by integral areas. Biological effects for normalized areas were estimated by calculating effect size. Additionally, they were analyzed via two-way ANOVA followed by Tukey’s post hoc test (Graph-Pad Prism 6 for Windows, Graph Pad Software, La Jolla, California, USA, www.graphpad.com, accessed on 15 December 2020).

### 2.6. Statistical Analysis

Results are expressed in mean (SD) ± standard error of the mean (SEM), median (Q1, Q3), or n (%). Normality was assessed using the Shapiro–Wilk test. Regarding the quantitative variables, the central tendency was compared using Student’s t or Mann–Whitney tests (2 categories) or ANOVA or the Kruskal–Wallis test (>2 categories). In qualitative variables, associations were verified using the Chi-squared test, with Fisher’s correction when necessary. Receiver operating characteristics (ROC) curves were calculated to assess the prognostic accuracy and determine the best cut-off points. A *p* < 0.05 indicated statistical significance. Statistical analysis was performed using SPSS 25.0 (SPSS, Chicago, IL, USA).

## 3. Results

### 3.1. Clinical Data

The baseline characteristics of healthy controls (*n* = 11) and biliary AP patients: mild AP (*n* = 19), moderately severe AP (*n* = 9), and severe AP (*n* = 10) are shown in Table 1. There were no significant differences in the demographic features between the control group and biliary AP patients. There were also no differences between the demographic characteristics in the three degrees of AP severity, as expressed in Table 1.

Several biochemical markers and multifactorial scores were assessed, both on admission and 48 h after onset of symptoms, which are expressed in Table 2.

Through the analysis of Table 2, both on admission and 48 h after onset of symptoms, all serological biomarkers except calcium (*p* = 0.82), albumin (*p* = 0.21), and total proteins (*p* = 0.34) on admission show significant changes between the different degrees of AP severity. All the multifactorial scores show significant changes between the degrees of severity of AP.

**Figure 1:**
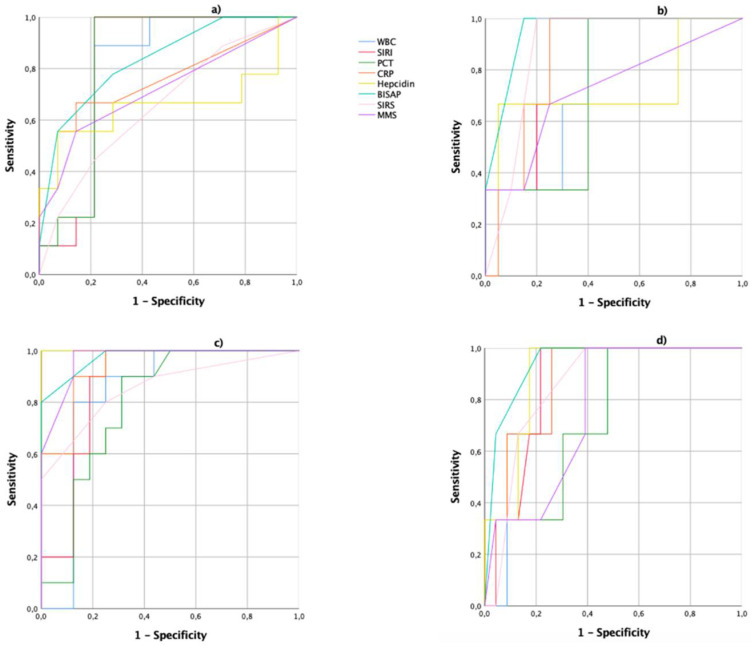
Receiver operating characteristics (ROC) curves for determining the cut-off values of WBC, SIRI, PCT, CRP, hepcidin, BISAP, SIRS, and MMS of (**a**) severe AP and (**b**) mortality on admission and (**c**) severe AP and (**d**) mortality 48 h after onset of symptoms. AP: acute pancreatitis; BISAP: bedside index for severity in acute pancreatitis; BMI: body mass index; CRP: C-reactive protein; MMS: modified Marshall score; SIRS: systemic inflammatory response syndrome; WBC: white blood cells.

On admission, PCT (0.83), BISAP (0.83), and SIRI (0.82) had the highest predictive values for severe AP. Regarding mortality, BISAP (0.95), SIRI (0.85), and PCR (0.85) had the highest predictive values. Forty-eight hours after onset of symptoms, for severe AP, the serological marker with the most significant predictive power was hepcidin (1.00) followed by BISAP (0.98), CRP (0.94), and SIRI (0.87). When analyzing the predictive power related to mortality, BISAP (0.94) was found to have the highest predictive value followed by hepcidin (0.90), CRP (0.88), and SIRI (0.86) as represented in Figure 1.

### 3.2. Patients with Biliary AP Presented Increased Levels of Plasma OS-Related Markers and Lower CAT and SOD Activities Than Healthy Subjects and Increased Levels of Lipid Peroxidation and Lower Levels of CAT and SOD in Severe Biliary AP

**Figure 2:**
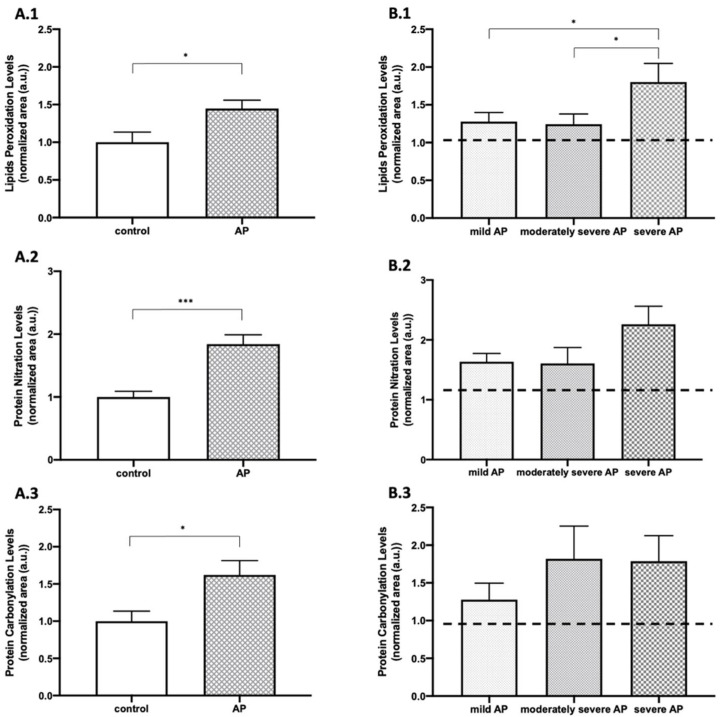
Plasma levels of OS-related markers of acute pancreatitis (AP) and healthy control groups. (**A.1**) 4-HNE (lipid peroxidation), (**A.2**) 3-NO (protein nitration), (**A.3**) DNP (protein carbonylation) in the control group and biliary AP patients. (**B.1**) 4-HNE (lipid peroxidation), (**B.2**) 3-NO (protein nitration), (**B.3**) DNP (protein carbonylation) in the different degrees of biliary AP severity on admission. AP: acute pancreatitis; 3-NO: 3-nitrotyrosine; 4-HNE: 4-hydroxynonenal; DNP: 2,4-dinitrophenyl-hydrazone. Results are expressed as mean ± SEM. The differences versus control are marked by *, where * represents *p* < 0.05 and *** *p* < 0.001. Mann–Whitney test: (**A.1**–**A.3**). Comparison of Fisher’s least significant difference post hoc test: (**B.1**–**B.3**).

As represented in Figure 2A, a significant increase on admission was verified related to the levels of lipid peroxidation (*p* < 0.05), protein nitration (*p* < 0.001), and protein carbonylation (*p* < 0.05) in AP patients when compared with healthy individuals. In addition, as shown in Figure 2B, there was a significant increase in lipid peroxidation when compared to mild and severe AP (*p* < 0.05) and moderately severe and severe AP (*p* < 0.05). A tendency for increased protein nitration levels in severe AP and in severe forms of AP in protein carbonylation, although without statistical significance, was also demonstrated.

**Figure 3:**
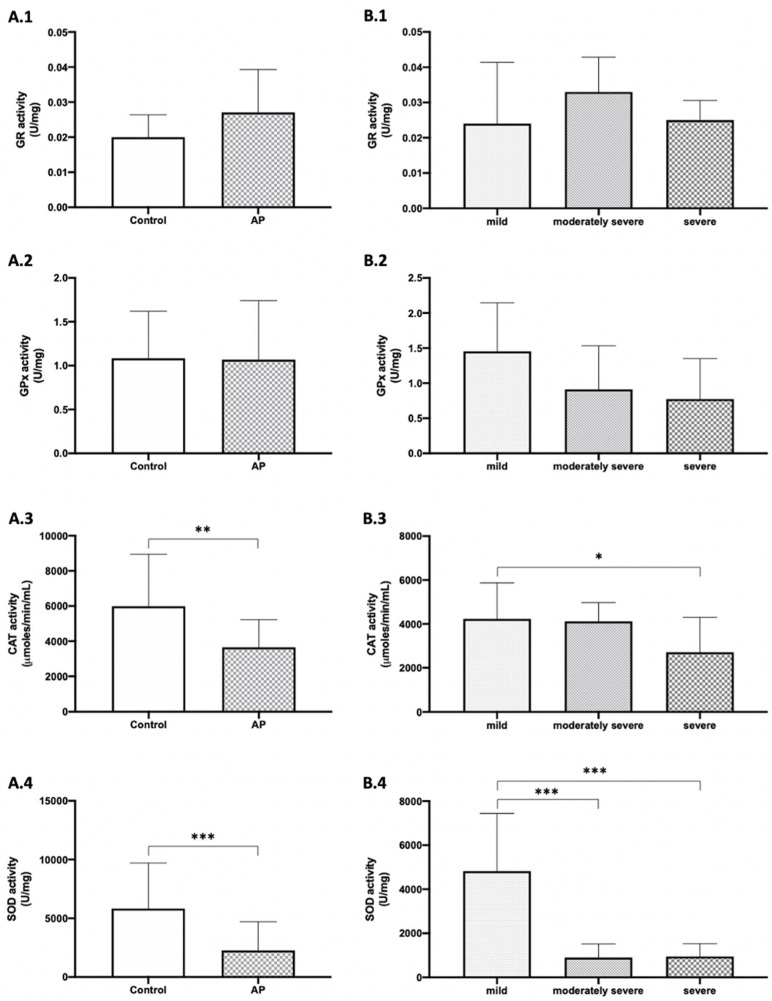
The activity of antioxidant enzymes in plasma of acute pancreatitis (AP) and healthy control groups. GR: glutathione reductase; GPx: glutathione peroxidase; CAT: catalase; SOD: superoxide dismutase from the healthy control group and biliary AP patients (**A**) and in the different degrees of biliary AP patients (**B**). Results are expressed as mean ± SEM. The differences versus control are marked by *, where * represents *p* < 0.05, ** *p* < 0.01 and *** *p* < 0.001. Mann–Whitney test: (**A.1**–**A.4**). Comparison of Fisher’s least significant difference post hoc test: (**B.1**–**B.4**).

The activity of enzymes related to the antioxidant system such as GR, GPx, CAT, and SOD were evaluated. As shown in Figure 3A, a tendency toward an increase in GR activity was detected in the plasma of AP patients compared to the healthy control individuals but without statistical significance. CAT and SOD presented with a significant decrease in AP patients’ activities compared to the healthy control group (*p* < 0.01 and *p* < 0.001, respectively).

According to the severity, as shown in Figure 3B, when analyzing the activity of the antioxidant enzymes in the plasma of patients with different degrees, there was a statistically significant decrease in CAT activity in patients with severe AP compared with mild biliary AP (*p* < 0.05). Plasma SOD activity was also significantly decreased when comparing patients with mild and moderately severe biliary AP and between mild and severe biliary AP patients (*p* < 0.001, for both).

### 3.3. NMR-Based Metabonomics Analysis of Plasma Samples from AP Patients

To analyze the changes in plasma metabolome related to pancreatic inflammation, we applied a nontargeted multivariate analysis. Both exploratory unsupervised method PCA and supervised method PLS-DA scores scatter plots of all severity degrees and analyzed at the same time revealed the significant influence of the inflammatory process on plasma metabolome composition, which can be observed in clustering trends of individual biliary AP groups.

**Figure 4:**
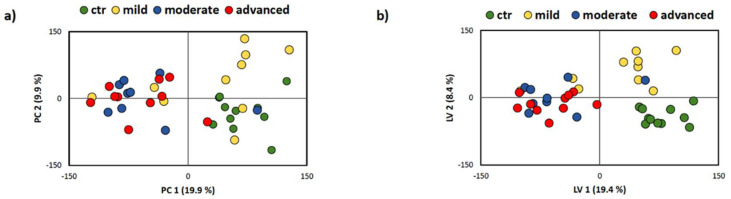
Scores scatter plots obtained via PCA (**a**) and PLS-DA (**b**) of 1D cpmg spectra of plasma from healthy control group and biliary AP patients. Degrees of biliary AP severity are indicated in the legends above the plots.

In Figure 4a, PCA was applied to test the internal data structure and search for clustering trends, possible group separation, and outliers. Obtained data demonstrated clustering according to tested groups. Overlap was observed between controls and mild biliary AP and moderately severe and severe biliary AP, indicating similarities of metabolomes. Additionally, data were analyzed by PLS-DA, as represented in Figure 4b, which is used to maximize the difference between groups.

**Figure 5:**
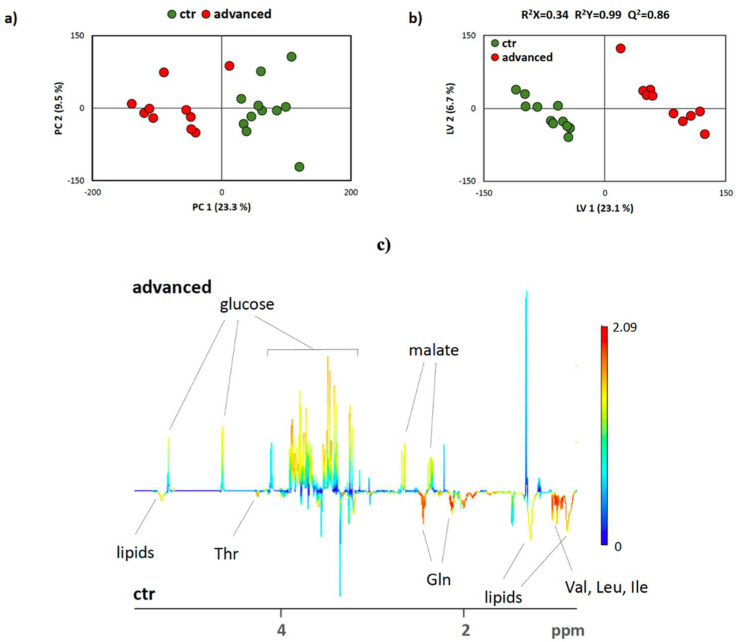
Scores scatter plots obtained via PCA (**a**) and PLS-DA (**b**) of 1D cpmg spectra of plasma from healthy control group and severe biliary AP patients. LV1 loadings extracted from PLS-DA are presented in (**c**). Loadings are colored according to variable importance of the projection (VIP), and some assignments are indicated.

Pair-wise comparisons between biliary AP severity degrees and the healthy control group were performed (PCA and PLS-DA) to identify the metabolites contributing to group separation. In PCA scores, scatter plot clustering according to tested groups and precise separation between the groups can be observed in Figure 5a. PLS-DA analysis further confirmed the groups’ separations. A sevenfold internal cross-validation was applied to assess the explained variance (R^2^) and predicative power (Q^2^) of PLS-DA models, as represented in Figure 5b. Although the internal validation yielded satisfactory model parameters, additional validation was performed via the permutation test.

**Figure 6:**
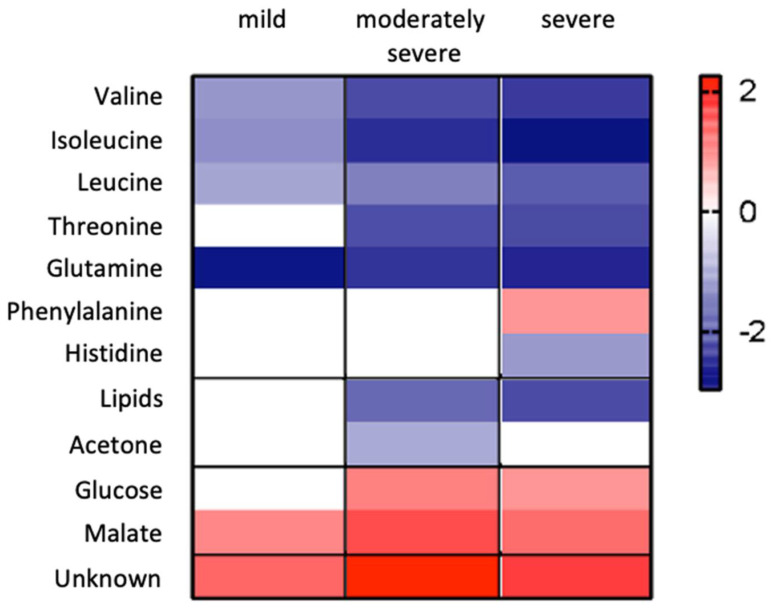
Heatmap of effect sizes representing plasma metabolome changes associated with severe biliary AP patients as compared with healthy control group.

The PLS-DA quality assessment parameters, goodness of fit to the data R^2^, and predictive value of the model Q^2^ were >0.8 in all the constructed models and corroborate model reliability and their suitability for data mining, as represented in Figure 5c. The results can also be presented as a heatmap (Figure 6) of biologically relevant effect sizes (ES > 0.7) [44] concerning the results attained in the plasma of the group of patients with biliary AP severity (mild, moderately severe, or severe) when compared with the healthy control group. This representation shows that plasma valine levels had an inferior difference between healthy and mild biliary AP, but it was gradually increased from moderately severe and severe AP. The same happens to isoleucine, threonine, histidine, and lipids contents. When phenylalanine was analyzed, there was a marked increase in healthy and severe AP levels. In turn, malate showed a marked increase between healthy and moderately severe AP patients.

### 3.4. Plasma Metabolic Profiles Show Differential Response to AP Diagnosis and Prognosis

**Figure 7:**
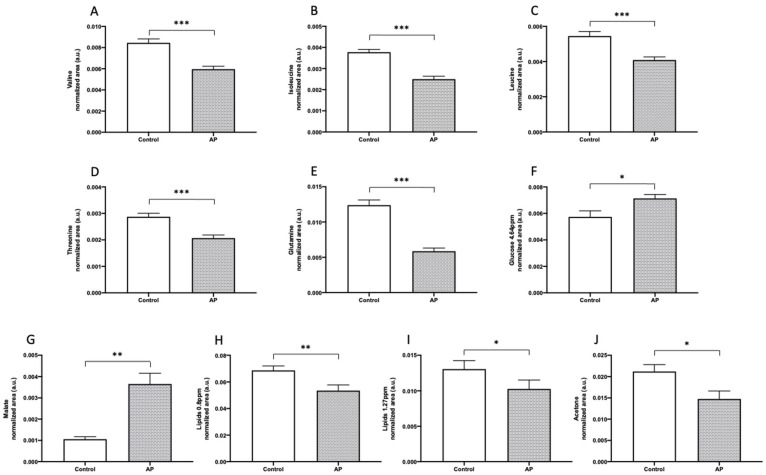
The quantitative abundance of different metabolites in the plasma of healthy control group and biliary AP patients. Results are expressed as mean ± SEM. The differences versus control are marked by *, where * represents *p* < 0.05, ** *p* < 0.01 and *** *p* < 0.001. Student’s *t* test: (**A**–**D**,**F**,**H**,**J**); Mann–Whitney test: (**E**,**G**,**I**).

The plasma metabolome seems to be highly sensitive to the diagnosis of AP. Among the different metabolites expressed in both groups, healthy control and biliary AP patients, were valine (0.009 ± 0.001 vs. 0.001 ± 0.002, *p* < 0.001), isoleucine (0.004 ± 0.001 vs. 0.003 ± 0.001, *p* < 0.001), leucine (0.005 ± 0.001 vs. 0.004 ± 0.001, *p* < 0.001), threonine (0.003 ± 0.0004 vs. 0.002 ± 0.001, *p* < 0.001), glutamine (0.012 ± 0.002 vs. 0.006 ± 0.003, *p* < 0.001), glucose (0.006 ± 0.002 vs. 0.007 ± 0.001, *p* < 0.05), malate (0.001 ± 0.0004 vs. 0.004 ± 0.003, *p* < 0.01), lipids (0.069 ± 0.01 vs. 0.054 ± 0.02, *p* < 0.05 and 0.013 ± 0.004 vs. 0.01 ± 0.007, *p* < 0.05), and acetone (0.021 ± 0.005 vs. 0.015 ± 0.01, *p* < 0.05), as represented in Figure 7.

**Figure 8:**
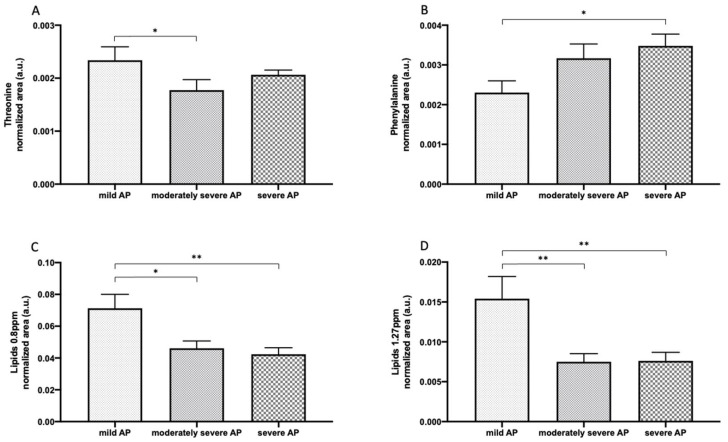
The quantitative abundance of different metabolites in the plasma of different degrees of biliary AP severity. Results are expressed as mean ± SEM. The differences versus control are marked by *, where * represents *p* < 0.05 and ** *p* < 0.01. Comparison of Fisher’s least significant difference post hoc test: (**A**–**C**); Comparison of the Tukey’s post hoc test: (**D**).

When plasma metabolomes were analyzed for AP prognosis, it was found that threonine (mild: 0.002 ± 0.001 vs. moderately severe: 0.002 ± 0.001, *p* < 0.05), phenylalanine (mild: 0.002 ± 0.001 vs. severe: 0.004 ± 0.001, *p* < 0.05), and lipids content (mild: 0.071 ± 0.028 vs. moderately severe: 0.046 ± 0.001, *p* < 0.05; mild: 0.071 ± 0.028 vs. severe: 0.054 ± 0.02, *p* < 0.01 and mild: 0.015 ± 0.009 vs. moderately severe: 0.008 ± 0.003, *p* < 0.01; mild: 0.015 ± 0.009 vs. severe: 0.01 ± 0.007, *p* < 0.01) showed changes with statistical significance, as represented in Figure 8.

## 4. Discussion

AP is an inflammatory disorder of the pancreas, responsible for many hospital admissions and associated with enormous economic cost [45,46]. In this sense, the stratification of the severity of AP is crucial not only for obtaining good clinical outcomes but also cost-effectiveness. The severity of AP can be predicted based upon clinical, laboratory, and radiologic risk factors, several score systems, and biomarkers. However, the complex mechanism of AP and its association with a progressive SIRS with high morbidity and mortality is not fully understood, and robust prognostic biomarkers are necessary [47,48]. Unfortunately, none has yet proven to be an excellent accurate predictor of the clinical course of AP. Gallstones are the leading cause of AP and result from the impaction of migratory gallstones in the ampulla. The population included in this study was exclusively composed of patients with biliary AP, considered the most common etiology in Mediterranean countries [8]. There are few studies aimed at this subtype of the patient population [49]. Currently, the diagnosis of AP is based on the RAC criteria that consider clinical, laboratory, and imaging factors [17]. The prognosis is based on several serological biomarkers and multifactorial scores. However, there is no specific prognostic tool for AP [13]. In this study, several serological biomarkers and multifactorial scores were analyzed. The times evaluated were on admission, considered the ideal time for assessment of prognosis, and 48 h after onset of symptoms to analyze the predictive power of the different markers when persistent organ failure was defined. Therefore, on admission, when the severity was assessed, it was found that the serological markers that had the most significant power were PCT and SIRI. When mortality was assessed, it was found that SIRI and CRP had the best predictive value. When severity and mortality were assessed 48 h after the onset of symptoms, it was found that hepcidin, CRP, and SIRI had the most significant predictive value.

PCT, a 116-amino-acid pro-peptide of calcitonin, was found to appear in high concentration during inflammation and sepsis, being released by hepatocytes, peripheral monocytes, and C-cells of the thyroid gland [50,51]. Increased PCT levels had been observed in severe AP, pancreatitis necrosis, and organ failure [46]. In the present study, on admission, PCT showed a better predictive value of severe AP (0.825).

SIRI was first described for the study of cancer-related inflammation [52]. This index lists the levels of neutrophils, monocytes, and lymphocytes in the blood count of cancer patients and their prognosis and in assessing the response of chemotherapy (QT) regimens [52,53]. Previous work demonstrated that this ratio also correlated with the severity of AP, and for the cut-off of 7.14, the sensitivity, specificity, and accuracy were 82%, 87%, and 85%, respectively. In the present study, this ratio proved to have good predictive power for the severity and mortality of AP on admission and 48 h after onset of symptoms.

CRP is an acute-phase protein synthesized by the liver, induced by cytokines like IL-6, and its level in the blood increases within hours in response to inflammation and sepsis. It was described as a predictive tool for AP prognosis and mortality [46]. This acute-phase reactant has been widely used as an independent predictor of AP severity and mortality, especially 48 h after hospital admission [54]. The same was verified in this study, showing an excellent predictive power for severity 48 h after the onset of symptoms. Upon admission, it proved to be a good predictor of mortality related to AP.

Hepcidin is a peptide hormone responsible for regulating the hemostasis of iron, which is the primary source of the synthesis of hepcidin in the liver. Extra-hepatic production of hepcidin was described in the heart, kidneys, retina, monocytes, and macrophages, alveolar cells, adipocytes, pancreatic β-cells, and bile [55]. The hepcidin levels increase during inflammation, primarily as a result of increased IL-6 and -1 [56]. Since hepcidin is also synthesized in the pancreas and biliary system, we can state that it predicts specific and nonspecific inflammation of AP, making it a unique biomarker. In the present study, as we showed in our previous study [13], hepcidin showed the best predictive power for both severity and mortality 48 h after onset of symptoms.

In the early events of AP, OS plays a pivotal role since ROS cause direct oxidative damage to lipids and proteins and modulate redox-sensitive transcription factors and redox-sensitive signal transduction pathways [57]. OS appears to have a relevant role in the initial phase of AP. Once produced, ROS can act as a molecular trigger for the pancreatic inflammatory process [58]. They also play a central role in perpetuating this process, leading to the progression of extrapancreatic complications [57,58]. In this study, on admission, plasma lipid peroxidation, protein nitration, and protein carbonylation were increased in biliary AP patients compared to the healthy control group. These results provided further evidence of the role of OS as a mediator in the initial phase of the pancreatic inflammatory process. The lipid peroxidation may be associated with primary cytosolic granules within polymorphonucleocytes and the inflammatory process [59]. Products of lipid peroxidation also may, in turn, be associated with damage of membrane integrity, inactivation of membrane-bound receptors, and enzymes, resulting in cell damage. These lipid peroxidation products, especially 4-HNE, react with proteins, changing their conformation and function, leading to enhanced inflammatory response [60]. Furthermore, OS-related markers of damage in proteins and lipids were studied in the plasma of patients with different degrees of biliary AP severity. The obtained data show statistically differences in the levels of lipid peroxidation between mild and severe biliary AP and moderately severe and severe biliary AP and no statistically relevant differences in protein nitration and protein carbonylation levels between the different degrees of biliary AP severity. However, it is essential to note that the small sample analyzed may influence these results and that a larger sample may show other results, namely statistical significance.

The inflammatory process in the pancreas has also been associated with a decline in antioxidant defenses, including GR, GPx, CAT, and SOD activities [61]. In the present study, CAT and SOD activities were significantly decreased in the plasma of patients with AP compared with the healthy control group. These enzymes are components of the cellular antioxidant enzyme system that detoxifies hydrogen peroxide and organic peroxides. Free radicals produced by active leukocytes may oxidize the active sites of these enzymes, thereby causing reduced activity. Of note, CAT and SOD showed decreases in plasma concentration of patients with different degrees of biliary AP severity. CAT activity in the plasma was further decreased in patients with severe biliary AP compared with those with mild biliary AP. SOD activity was also decreased in the plasma of patients with severe biliary AP. These findings are consistent with previous studies, which reported a decrease in antioxidant enzymes’ expression in murine models and patients with AP [62,63]. In addition, the decrease of antioxidant defenses may explain the increase in lipid peroxidation among patients with severe biliary AP. Thus, the obtained data further support that the different degrees of AP severity are associated with different degrees of OS-related effects, being that severe AP induces major lipid peroxidation as detected in the plasma. Thus, those patients present a lower antioxidant capacity presented as decreased activity of antioxidant enzymes. However, further studies will be needed to unveil whether that is a cause or a consequence of severe AP.

Metabonomics is a rapid and noninvasive analysis that consists of the systematic study of metabolites as small-molecule biomarkers that represent the functional phenotype in a cell, tissue or organism [64]. The obtained data suggested metabolic dysfunction, and, thus, we applied a metabonomic-based NMR study to metabolically characterize the plasma of patients with different degrees of biliary AP severity. On admission, valine, isoleucine, leucine, threonine, glutamine, glucose, malate, and acetone were significantly altered in AP patients’ plasma compared with the healthy control group, highlighting that, patients with AP present a systemic alteration in their metabolic profile. Regarding the prognosis of biliary AP severity, threonine and phenylalanine showed changes in the plasma of patients with mild and moderately severe biliary AP and mild and severe biliary AP, respectively, while lipids content was changed in the plasma of patients with mild and moderately severe biliary AP and mild and severe AP. These findings demonstrate that biliary AP causes evident disruption of pancreatic metabolism at a molecular level, and these metabolites could be potential markers for the diagnosis or onset and prognosis of biliary AP. The characterization of the metabonomic profile in the present study allowed for the identification of metabolites affected by biliary AP severity.

Branched-chain amino acids signals (valine, leucine, and isoleucine) were decreased in the plasma of patients with biliary AP compared to the control group. This observation was also described by Villaseñor et al. [27]. It is very relevant because these amino acids are a component of pancreatic juice, and the decrease in their concentration in circulation may be related to the exocrine pancreatic insufficiency characteristics of severe AP [65]. Thus, they should be regarded as potential biomarkers for the onset and with the potential to determine the progression of AP, but more studies will be needed to validate these hypotheses.

Glutamine is a crucial element in the tricarboxylic acid cycle [66]. During catabolic stress, namely sepsis, its levels rapidly decrease [67]. Glutamine is also important since it maintains gut barrier function [68], which prevents the progression of inflammation and infection in AP. In this study, plasma glutamine concentration was decreased in the patients with biliary AP, suggesting that it inhibits the metabolization of this substrate by repressing the metabolization of glutamine at some level. Since the gut barrier can be affected, there may be a progression of the inflammatory process, namely in the necrosis infection verified in the severe AP.

Malate concentration in the plasma was also altered when comparing to the healthy control group to biliary AP patients. This metabolite is a product of carbohydrate breakdown through the tricarboxylic acid cycle to provide additional energy in the form of adenosine phosphate [69]. The increase in plasma concentration of malate detected in biliary AP patients suggests an increased demand for energy due to the severe acute pancreatic and systemic inflammatory process. This study demonstrated an increase of malate in the plasma of patients with all degrees of biliary AP severity, suggesting a possible involvement of the tricarboxylic acid cycle in the inflammatory process mediated by the onset of biliary AP.

In this study, low plasma levels of threonine were found in patients with biliary AP compared to the healthy control group and in moderately severe biliary AP patients when compared with mild biliary AP patients. Low levels of threonine are associated with changes in inflammatory cytokines [70]. This action is more accentuated in the intestinal immune response. In AP, changes in intestinal immunity may be associated with bacterial translocation and the presence of secondary pancreatic infection, which occurs mainly in the severe form of AP.

Phenylalanine is an amino acid strongly associated with mortality in patients with severe infection since high levels are related to severe metabolic disturbance [71]. In this study, the plasma levels of phenylalanine were higher in the patients with severe biliary AP than the levels detected in patients with mild biliary AP. Phenylalanine is one of the essential amino acids that can be oxidized into tyrosine via hydroxylase catalysis and together with tyrosine to synthesize important neurotransmitters and hormones. Overall, taking into consideration our data, this amino acid may be considered as a prognostic marker for severe biliary AP.

Lipid metabolism can cause OS reaction and acidosis, leading to injury of the pancreas, surrounding tissues, and hypoxic necrosis, features of the severe form of AP [72]. AP could lead to abnormal lipid metabolism under the regulation of nerves and body fluids [73].

This study had several limitations that must be considered when interpreting the data. The most important being the relatively small number of patients. However, we detected crucial changes in the metabonomics and OS analyses of plasma of patients with different degrees of biliary AP severity. Some may be used as biomarkers for the onset and progression of AP but need further validation with a higher number of samples.

## 5. Conclusions

This study allowed us to confirm the role of hepcidin and SIRI as new prognostic biomarkers of biliary AP. Overall, our results confirmed that OS has a role in the onset and progression of the pancreatic inflammatory process. This study also constituted the first metabonomic study in the field of biliary AP, and it allowed us to show that metabonomics can be fundamental for understanding the pathophysiology of AP. We identified molecular mechanisms associated with metabolic and OS profiles that may predict the onset and progression of biliary AP severity. This study may help identify and better understand mechanisms underlying the pancreatic inflammatory process, allowing for the eventual development of new and relevant target therapies.

## Figures and Tables

**Table 1:** Baseline characteristics of healthy controls and biliary AP patients included in the study. (^‡^ Mann–Whitney test; ^‡‡^ Chi-squared test; ^‡‡‡^ Kruskal–Wallis test).

	Control (*n* = 11)	Sample (*n* = 29)	*p*	Mild AP (*n* = 10)	Moderately Severe AP (*n* = 9)	Severe AP (*n* = 10)	*p*
Age (years)	69 ± 14.6	65 ± 18.9	0.65 ^‡^	54 ± 16.8	69 ± 17.2	73 ± 18.7	0.53 ^‡‡‡^
Female sex	6 (54.5%)	20 (69%)	0.41 ^‡‡^	8 (80%)	6 (66.7%)	6 (60%)	0.39 ^‡‡^
BMI (kg/m^2^)	26.7 ± 3.0	26.7 ± 3.8	0.98 ^‡^	27.0 ± 2.9	26.4 ± 4.6	26.6 ± 4.0	0.92 ^‡‡‡^
BMI ≥ 30 kg/m^2^	2 (18.2%)	6 (20.7%)	0.91 ^‡‡^	2 (20%)	2 (22.2%)	2 (20%)	0.91 ^‡‡^
CCI	3 (1–4)	3 (1–4)	0.49 ^‡^	1 (0–2)	3 (2–4)	3 (1–4)	0.49 ^‡‡‡^
Hospital stay (days)		12 ± 7.8	-	6 ± 2.5	11 ± 5.0	18 ± 8.7	-
Mortality		3 (10.3%)				3 (30%)	

AP: acute pancreatitis; BMI: body mass index; CCI: Charlson comorbidities index.

**Table 2:** Several biochemical markers and multifactorial scores of healthy controls and biliary AP patients included in the study. (^‡^ Kruskal–Wallis test).

	Controls (*n* = 11)	Time	Sample (*n* = 29)	Mild AP (*n* = 10)	Moderately Severe AP (*n* = 9)	Severe AP (*n* = 10)	*p* ^‡^
WBC	6.5 ± 1.6	Admission	14.5 ± 5.4	11.2 ± 4.5	15.0 ± 5.6	17.4 ± 4.1	0.017
		48 h	12.1 ± 6.3	6.9 ± 3.5	12.7 ± 7.5	14.9 ± 2.8	0.002
Neutrophils	4.8 ± 0.9	Admission	12.4 ± 5.5	8.6 ± 4.5	13.2 ± 5.3	15.7 ± 3.6	0.011
		48 h	10.0 ± 6.2	4.7 ± 3.2	10.4 ± 6.9	13.2 ± 2.7	0.001
SIRI		Aission	7.4 ± 6.3	3.6 ± 3.9	8.8 ± 7.5	11.4 ± 4.9	0.022
		48 h	6.6 ± 6.4	1.6 ± 2.3	5.9 ± 6.4	10.6 ± 5.7	0.001
PCT		Admission	2.9 ± 6.9	0.7 ± 0.9	4.5 ± 5.8	5.9 ± 10.9	0.001
		48 h	4.1 ± 7.3	0.6 ± 0.1	6.6 ± 9.1	7.9 ± 8.8	<0.001
CRP	5.3 ± 1.1	Admission	42.8 ± 82.7	5.4 ± 1.2	33.0 ± 51.5	88.9 ± 22.0	0.032
		48 h	170.7 ± 152.6	15.9 ± 14.0	180.6 ± 107.2	316.6 ± 107.9	<0.001
Hepcidin		Admission	64.2 ± 69.6	20.4 ± 12.2	69.9 ± 37.9	100.7 ± 90.5	0.047
		48 h	136.7 ± 138.7	26.4 ± 27.2	56.6 ± 51.9	286.7 ± 87.3	<0.001
Calcium		Admission	9.0 ± 0.6	8.9 ± 0.5	9.1 ± 0.4	8.9 ± 0.9	NS
		48 h	8.5 ± 0.8	8.9 ± 0.3	8.7 ± 0.5	7.9 ± 1.0	0.014
Albumin		Admission	3.7 ± 0.5	3.8 ± 0.4	3.7 ± 0.4	3.5 ± 0.5	NS
		48 h	3.1 ± 0.5	3.5 ± 0.3	3.2 ± 0.3	2.7 ± 0.6	0.001
Total proteins		Admission	6.5 ± 0.6	6.7 ± 0.5	6.7 ± 0.5	6.3 ± 0.6	NS
		48 h	5.9 ± 0.6	6.4 ± 0.4	6.0 ± 0.3	5.3 ± 0.6	0.001
BISAP		Admission	1 (1–3)	1 (0–1)	1 (1–2)	3 (2–3)	<0.001
		48 h	2 (1–4)	1 (0–1)	1 (1–3)	4 (4–5)	<0.001
SIRS		Admission	1 (1–2)	1 (0–1)	2 (1–2)	2 (1–2)	0.045
		48 h	1 (0–2)	0 (0–1)	1 (0–2)	3 (2–3)	0.001
MMS		Admission	0 (0–1)	0 (0–0)	0 (0–2)	1 (0–2)	0.017
		48 h	0 (0–2)	0 (0–0)	0 (0–2)	3 (2–3)	<0.001

AP: acute pancreatitis; BISAP: bedside index for severity in acute pancreatitis; BMI: body mass index; CRP: C-reactive protein; NS: not significant; MMS: modified Marshall score; SIRS: systemic inflammatory response syndrome; WBC: white blood cells.

## Data Availability

All generated data in this study are included in the article.
